# Physical Activity as a Central Pillar of Lifestyle Modification in the Management of Chronic Musculoskeletal Pain: A Narrative Review

**DOI:** 10.3390/jfmk10020183

**Published:** 2025-05-20

**Authors:** Rodrigo Núñez-Cortés, Joaquín Salazar-Méndez, Jo Nijs

**Affiliations:** 1 Department of Physical Therapy, Faculty of Medicine, University of Chile, Santiago 8240000, Chile; r_nunez@uchile.cl; 2 Escuela de Ciencias del Deporte y Actividad Física, Facultad de Salud, Universidad Santo Tomás, Talca 3460000, Chile; 3 Pain in Motion Research Group (PAIN), Department of Physical Therapy, Human Physiology and Anatomy, Faculty of Physical Education & Physical Therapy, Vrije Universiteit Brussel, 1090 Brussels, Belgium; 4 Chronic Pain Rehabilitation, University Hospital Brussels, 1090 Brussels, Belgium; 5 Department of Health and Rehabilitation, Unit of Physiotherapy, Institute of Neuroscience and Physiology, Sahlgrenska Academy, University of Gothenburg, 405 30 Gothenburg, Sweden

**Keywords:** exercise, chronic pain, lifestyles, sleep, diet, stress

## Abstract

**Objective**: This narrative review aims to analyze physical activity as a central pillar of lifestyle modification in the management of chronic musculoskeletal pain by examining its effects on pain modulation as well as related lifestyle domains, including sleep, stress regulation, dietary habits, and smoking behavior. **Methods**: A narrative structured review was conducted. We searched MEDLINE/PubMed, Embase, and Cochrane Reviews using terms related to chronic pain and lifestyle. Randomized controlled trials, observational studies, systematic reviews, and narrative reviews reporting on the concepts of interest were included. The results were synthesized and described narratively. **Results**: Through the release of neuromodulatory compounds such as endorphins, endocannabinoids, dopamine, and serotonin, exercise improves analgesia, promotes emotional resilience, and reduces the reward response associated with addictive behaviors such as smoking. Its effects on the hypothalamic–pituitary–adrenal axis reduce cortisol levels, while melatonin regulation promotes circadian synchronization and deeper sleep stages. In addition, exercise modulates appetite by increasing insulin sensitivity and altering hormones such as leptin and ghrelin, contributing to appetite control and energy balance. These mechanisms support a comprehensive approach to chronic pain management. **Conclusions**: Physical activity is a core component of lifestyle-based chronic pain management, not only because of its analgesic effects, but also because of its positive influence on sleep, stress regulation, dietary habits, and smoking reduction. Although the available evidence is promising, more randomized controlled trials are needed to examine the effects of exercise on other healthy lifestyle behaviors, such as stress reduction, dietary modification, and smoking cessation, to consolidate its role in the comprehensive prevention and management of chronic pain.

## 1. Introduction

Chronic pain is a major global health problem, affecting more than 30% of the world’s population and causing economic costs comparable to those of cancer and cardiovascular disease [1,2]. This condition is the leading cause of disability worldwide, profoundly affecting daily activities, social interactions, quality of life, and work capacity [3]. Evidence has also shown that people with chronic widespread pain experience excess mortality [4]. The excess mortality observed in people with chronic pain could be largely explained by modifiable lifestyle factors, reinforcing the need for interventions aimed at improving physical activity, diet, weight control, and smoking cessation in this population [4].

Chronic pain is defined as pain that persists longer than the normal tissue healing time, usually considered to be 12 weeks [5], and is characterized by functional and structural brain changes, neuroinflammation, and central sensitization [6,7]. Emerging evidence also highlights the role of lifestyle factors—such as physical inactivity, stress, sleep problems, unhealthy diet, and smoking—in influencing the severity and persistence of chronic pain [8]. These findings support a paradigm shift toward integrative, lifestyle-based approaches to chronic pain management that address both its symptom burden and long-term health risks.

Advances in the understanding of chronic pain in recent decades have led to a significant shift in therapeutic approaches away from exclusively biomedical interventions towards multimodal strategies that integrate the biopsychosocial complexity of chronic pain [9]. This shift also promotes self-management and patient autonomy through active treatments [10]. In this regard, physical activity and exercise are recognized as essential components in the treatment of chronic pain, due to their safety and demonstrated clinical benefits in improving physical and mental health [11,12,13,14]. Beyond its direct benefits, physical activity can also influence the full spectrum of lifestyle factors, supporting not only pain relief but also broader improvements in overall lifestyle.

Chronic pain is a major global health challenge and a leading cause of disability worldwide. While its clinical management has evolved toward multimodal strategies, current approaches still often overlook the interrelated role of lifestyle behaviors (sleep, stress, diet, and smoking) in shaping the pain experience. Despite the wealth of evidence supporting physical activity as a safe and effective treatment for chronic pain, its potential as a central modulator of other health-related behaviors remains underexplored. A clearer understanding of this relationship could inform more holistic and integrated rehabilitation strategies. The aim of this narrative review is to analyze physical activity as a central pillar of lifestyle modification in the management of chronic musculoskeletal pain by examining its effects on pain modulation as well as related lifestyle domains, including sleep, stress regulation, dietary habits, and smoking behavior.

## 2. Materials and Methods

This study was a narrative review using an evidence search and selection approach. MEDLINE/PubMed, Embase, and the Cochrane Reviews were searched from inception to 19 November 2024. Specific search terms included combinations of “chronic pain”, “physical activity”, “exercise”, “sleep”, “stress”, “diet”, “nutrition”, and “smoking”, linked by Boolean operators (AND/OR). The search strategy is presented in Table A1 (Appendix A). Additional records were identified through manual searches, including reference lists of eligible articles and related reviews. No date or language restrictions were imposed. Two independent reviewers were involved in the selection of studies, and disagreements were resolved by consensus. Rayyan software (http://rayyan.qcri.org) was used for duplicate removal and study selection.

The research team selected relevant studies that exemplified the most relevant information on the topic. We included randomized controlled trials, systematic reviews, and meta-analyses that reported on the concepts of interest. Eligibility was determined based on thematic relevance to the predefined areas of interest and the clarity of reported outcomes. We preferred primary studies that directly investigated the concepts of interest and systematic reviews that summarized the evidence. If two studies reported similar information, the more recent and complete study was included in the manuscript. Commentaries, letters to the editor, protocols, and trial registries were excluded from this review.

All information gathered in this review was summarized and qualitatively classified according to an outline previously defined by the team: (1) physical activity and modulation of chronic pain, (2) physical activity and improvement of sleep quality, (3) physical activity to reduce stress, (4) impact on diet and metabolic regulation, (5) physical activity and smoking reduction. This framework was established a priori by the team based on known associations between physical activity and lifestyle factors that influence chronic pain. The information provided by the studies was summarized and synthesized narratively and is presented in figures as a summary (created with BioRender.com). Furthermore, a standardized table was created to extract information from the representative studies, including the following data: study design, population, lifestyle domain, and main findings.

## 3. Results

**Figure A1:**
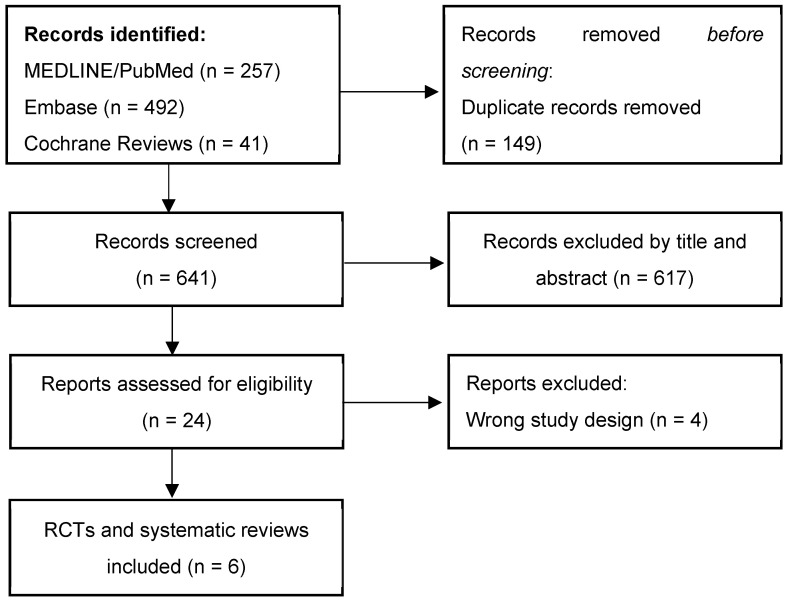
Study selection process.

Figure A1 (Appendix A) illustrates the selection process for the main RCTs and systematic reviews retrieved through the structured database search. Other relevant articles selected for their thematic relevance are included in the narrative synthesis.

### 3.1. Physical Activity and Modulation of Chronic Pain

Physical activity, which includes different forms such as aerobic exercise and resistance training, modulates pain pathways through peripheral and central mechanisms. Exercise stimulates the production of neurotransmitters and neurotrophic factors, such as brain-derived neurotrophic factor (BDNF), in brain areas involved in pain perception, such as the hippocampus and prefrontal cortex [15,16,17], favoring activity-dependent neuronal plasticity [15,18] and emotional regulation [19]. Chronic pain, often characterized by human-assumed central sensitization, involves an amplification of nociceptive signals in the central nervous system [6,7]. In this sense, physical activity decreases the release of excitatory neurotransmitters, such as glutamate, and increases the production of endogenous opioids (endorphins and enkephalins) and endocannabinoids, which reduce pain sensitivity through their actions on the pathways of the central and peripheral nervous systems [20]. Also, regular physical activity may have a protective effect on the brain by promoting the release of serotonin and BDNF, which may both play key roles as pain modulators [21,22]. Animal studies suggest that regular aerobic exercise increases serotonin release and modulates synaptic plasticity in the anterior cingulate cortex, a region essential for pain processing [21]. Exercise-induced analgesia is explained by the activation of central inhibitory pathways, mediated by opioid and serotonergic mechanisms and the modulation of NMDA receptors in the rostral ventromedial medulla [23], which regulate nociceptive transmission and decrease pain perception.

**Figure 1:**
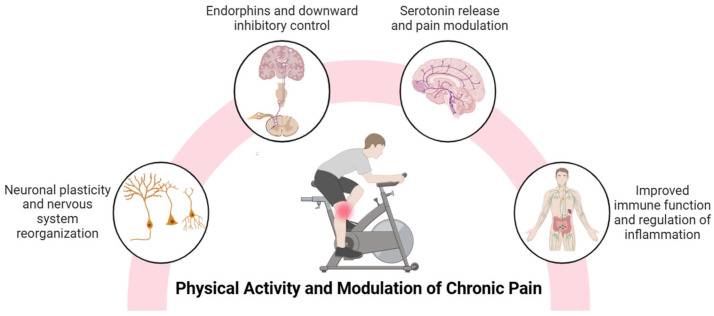
A summary of the benefits of physical activity in the modulation of chronic pain.

Central sensitization involves the activation of inflammatory cells such as macrophages and the release of pro-inflammatory mediators, including cytokines and chemokines, in both the peripheral and central nervous systems. In the central nervous system, this process is closely associated with neuroinflammation, which is characterized by the activation of glial cells such as microglia and astrocytes in the spinal cord and brain [24]. Studies have shown that glial density is increased in the brains of patients with chronic non-specific low back pain, migraine, and fibromyalgia, supporting the role of neuroinflammation in the development of chronic pain [25,26]. Inflammatory markers interact with nociceptors and spinal cord neurons, leading to altered excitability, conductance and transmission in pain pathways, disrupting their normal processing [27] and playing a critical role as mediators of neuroinflammation associated with chronic pain [28,29]. Recent studies identify an imbalance in cytokine levels in fibromyalgia patients, characterized by elevated tumor necrosis factor-alpha (TNF-α), interleukin-6 (IL-6), and interleukin-8 (IL-8) [30], with serum concentrations of IL-6 and IL-8 significantly correlating with disease severity [31,32]. Likewise, in patients with knee osteoarthritis, increased levels of intra-articular biomarkers of acute inflammation are related to peripheral sensitization, while biomarkers of cartilage degeneration and chronic inflammation are associated with central sensitization [33]. In this context, physical activity can improve the function of the immune system, which plays an essential role in the regulation of inflammation [34]. Exercise impacts the immune system through the release of exercins, signaling molecules produced by various tissues in the body [35]. Physical activity may induce circulatory and intra-articular anti-inflammatory effects in patients with knee osteoarthritis [22], as well as inducing an immunomodulatory response in fibromyalgia, characterized by decreased pro-inflammatory signaling, especially IL-8 [36]. Finally, exercise improves mitochondrial function and reduces oxidative stress [37,38], key factors in the maintenance of chronic inflammation. The main mechanisms by which physical activity can mitigate chronic pain are summarized in Figure 1. It is important to note that some of these mechanisms are supported by preclinical studies and may not be fully established in chronic pain populations.

### 3.2. Physical Activity and Improvement of Sleep Quality

Chronic musculoskeletal pain and sleep problems have a bidirectional relationship [39,40], meaning that they both reinforce each other and influence symptomatology through psychological, physiological, and attentional factors [41]. A recent meta-analysis reported that up to 75% of people with chronic musculoskeletal pain have sleep problems [42]. The high prevalence of sleep disturbances in these individuals could be related to common physiological mechanisms, such as the possible modulation of endogenous substances, altered melatonin levels, low-grade systemic inflammation that sensitizes the nociceptive system, and the possible alteration of the circadian rhythm [43,44,45,46,47,48]. Therefore, sleep problems should be addressed in the management of persons with chronic musculoskeletal pain who have a comorbid sleep disorder [49].

Physical activity has been proposed as an effective alternative to improve sleep problems in people with chronic musculoskeletal pain [50,51,52]. Recent meta-analyses confirm that different types of exercise (e.g., aerobic exercise, resistance training, stabilization exercise, and mind–body exercise) significantly improve sleep in this population, but effect sizes are small and may not reach the level of clinical significant improvements [53,54,55]. The small beneficial effect is explained by factors such as increased central physiological fatigue, exercise-induced analgesia, and improved psychological function, including mood and reduced anxiety [56,57,58]. In addition, exercise regulates key biological processes, such as decreasing pro-inflammatory cytokines [59], BDNF [60], and serotonin secretion [61], which play important roles in regulating wakefulness and rapid eye movement (REM) sleep [55,62,63]. It also adjusts the circadian rhythm through the release of melatonin (modulated by serotonin) [64,65], a hormone that regulates the onset, maintenance, and quality of sleep [66]. Nevertheless, exercise performed in the afternoon tends to alter the circadian rhythm in healthy individuals [67]. Therefore, careful consideration should be given to the timing of exercise. On the other hand, endorphins and endocannabinoids released during exercise promote relaxation and reduce stress [68] by acting on receptors in the central nervous system, regulating pain perception and mood, which facilitates sleep onset and sleep quality [68,69]. Moreover, exercise modifies sleep architecture by increasing the proportion of deep sleep (N3 phase) in healthy people, a crucial stage for physical and mental recovery [70]. The effect of exercise on sleep architecture, especially in increasing deep sleep, requires further investigation in people with chronic pain.

Research suggests that nocturnal exercise generally does not adversely affect sleep in healthy individuals [71,72,73]. However, performing high-intensity exercise close to bedtime may alter some components of sleep [73]. In people with severe chronic pain, it has been proposed that high-intensity physical activity during leisure time, regardless of the time of day, may increase the risk of sleep disturbance [74]. Therefore, future studies should investigate the most appropriate frequency, intensity, duration, timing, and type of therapeutic exercise for different clinical conditions, since the current evidence is still limited in these aspects [55]. In any case, it is clear that the beneficial effects of exercise therapy on sleep in patients with chronic pain with sleep problems (e.g., insomnia, sleep apnea) are not large enough to have a clinically meaningful impact on sleep outcomes. Hence, despite the small beneficial effects of exercise therapy on sleep in patients with chronic pain, specific sleep treatment is mandatory to ‘solve’ the sleep problem. This often implies combining exercise therapy with the first-line evidence-based treatment for insomnia and most sleep problems: cognitive–behavioral therapy for insomnia. A recent study supports the combined used of cognitive–behavioral therapy for insomnia with cognition-targeted exercise therapy and pain science education in patients with chronic spinal pain with comorbid insomnia [75]. In that study, sleep treatment was initiated early in the treatment, aligning well with the idea that patients require proper sleep to be able to recover from exercise or physical activity interventions. Sleep might be a prerequisite for optimal exercise effects.

### 3.3. Physical Activity to Reduce Stress

Stress represents an organism’s efforts to maintain homeostasis. In chronic pain patients, stress intolerance (due to physical, psychosocial, or emotional stressors) exacerbates symptoms such as pain, fatigue, and cognitive impairment [76]. A dysfunctional stress system in chronic pain patients is characterized by an imbalance between the sympathetic and parasympathetic branches of the autonomic nervous system, with sympathetic predominance keeping the body in a constant state of physical stress [76,77]. In addition, the hypothalamic–pituitary–adrenal (HPA) axis, which plays a key role in stress recovery and has metabolic and immunoregulatory functions, can be dysfunctional, ranging from hypercortisolism to hypocortisolism [78,79]. Thus, stress intolerance implies that the body is physiologically unable to cope effectively with stressors.

Physical activity could be a key strategy for improving stress tolerance in chronic pain patients. Regular exercise reduces resting sympathetic tone and basal cortisol levels, improving stress regulation [80]. In healthy subjects, muscle-strengthening physical activity moderately stimulates the HPA axis, reducing the resting cortisol response and inflammation [81]. Importantly, chronically elevated cortisol is associated with increased cardiovascular risk [82]. This mechanism could also apply to people with chronic pain, a hypothesis that merits further investigation. Exercise also decreases systemic inflammation and oxidative stress, two factors that aggravate the stress response. This protects the brain against the detrimental effects of chronic stress [83]. Additionally, neurotransmitters (serotonin, endocannabinoids, and endogenous opioids) released during exercise produce a sense of well-being and contribute to emotional resilience [83].

Despite the above, few clinical trials have evaluated the effect of exercise on perceived stress in people with chronic pain. One randomized controlled trial found that Pilates exercise can effectively decrease the level of perceived stress and pain intensity in women affected by premenstrual syndrome [84]. Another randomized controlled trial study reported that a walking program improved perceived stress levels in patients with chronic low back pain [85]. According to the secondary analysis of one randomized controlled trial, high-intensity training has also been shown to decrease both central sensitization and perceived stress in people with non-specific chronic low back pain [86]. Importantly, stress reduction could be maximized if physical activity interventions are performed with exposure to green spaces [87], and in groups, taking into account the benefits of social support for stress management in people with chronic pain [79]. Stress management is critical for people with chronic pain, as a dysfunctional stress response system perpetuates chronic pain. In this context, physical activity, through its physiological effects, is positioned as a fundamental tool to promote resilience to stress.

On the other hand, it has been suggested that patients with chronic pain could benefit from a stress management program in preparation for exercise therapy [88]. Any exercise bout or physical activity is a (healthy) stressor, implying that improved stress tolerance will facilitate patients to cope better with (incremental) exercise programs, including dealing with possible pain flares or any other temporary discomfort associated with exercise programs or physical activity. In addition, many patients with chronic pain present with overactivity/persistence behavior [89,90] due to motivational or contextual factors related to their perceived roles in life and in spite of experiencing frequent pain flares during and in response to these activities. Such persisted activities and exercises typically increase stress levels in patients with chronic pain, requiring skills to accept and cope better with the stress rather than (graded) exercise therapy programs.

### 3.4. Impact on Diet and Metabolic Regulation

People with persistent pain often have unhealthy dietary habits (e.g., low fruit and vegetable intake and pro-inflammatory dietary behaviors) that can negatively affect pain management. For example, deficiencies in essential nutrients such as antioxidants, omega-3 fatty acids, vitamin D, and magnesium appear to be strongly associated with pain [91,92]. Recent studies suggest a causal relationship between higher intakes of fresh and dried fruits and grains and lower pain scores, while high intakes of salt, alcohol, poultry, and pork are associated with higher persistent pain scores [93]. Chronic systemic and low-grade inflammation, associated with diets poor in antioxidants and anti-inflammatory agents, elevates biomarkers such as C-reactive protein, triggering neuroinflammation and nerve sensitization that favor the chronification of pain [94,95]. Also, deficiency of neurotransmitter precursors, such as tryptophan for serotonin, aggravates pain sensitivity [96]. In this context, chronic pain, especially high-intensity pain, is independently associated with dyslipidemia, obesity, a high waist-to-hip ratio, increased cardiovascular risk, and an increased prevalence of metabolic syndrome [97].

Physical activity may be an appropriate strategy for improving eating habits. Exercise influences energy balance not only by increasing calorie expenditure, but also by modifying appetite control through physiological and psychological mechanisms. This effect includes changes in hormones such as ghrelin, leptin, and insulin, and gastrointestinal peptides such as GLP-1 and CCK, which regulate hunger and satiety [98]. Furthermore, fat mass, fat-free mass, and resting metabolic rate play essential roles in the expression of appetite, with fat-free mass acting as a possible appetite signal derived from skeletal tissue [98,99]. On the other hand, sedentary lifestyles are associated with increased adiposity, overconsumption, and appetite dysregulation, possibly mediated by molecular signals that are not yet fully understood [99].

Physical activity not only promotes weight control and reduces metabolic inflammation, but also improves insulin sensitivity [100], counteracting the metabolic dysfunction common in people with chronic pain [97]. Additionally, exercise may act as a modulator between dopamine and the reward system [101], helping to regulate appetite, reduce anxiety, and promote healthier eating habits. Exercise improves eating habits by reducing emotional eating, increasing self-efficacy, and promoting self-regulation, facilitating better control of caloric intake and conscious dietary choices [102]. This link highlights the importance of exercise not only for energy balance, but also for the emotional and behavioral regulation associated with eating. In this regard, an 18-month randomized clinical trial of 454 overweight or obese older adults with osteoarthritis of the knee found that the combination of diet and exercise resulted in greater weight loss (11.4%), lower IL-6 levels, and improvements in pain, function, and quality of life compared with diet or exercise alone [103]. These findings highlight the benefits of integrated approaches to treating musculoskeletal conditions in overweight and obese adults. In fact, individualized nutritional interventions improve the management of persistent pain by focusing on healthy eating patterns (vegan, vegetarian, Mediterranean) for their anti-inflammatory properties and gut benefits [104]. Finally, the dietary intake of sufficient nutrients is essential for allowing the human body to exercise, suggesting that dietary interventions should accompany or even precede exercise therapy for patients with chronic pain. For the same reason, in non-chronic pain populations, dietary strategies are becoming increasingly recognized as potential strategies to optimize training effects and recovery from exercise interventions [105], creating important innovative angles for future research in the field of exercise therapy in patients with chronic pain.

### 3.5. Physical Activity and Smoking Reduction

Over their lifetime, people with chronic pain are more likely to be active smokers and to have been diagnosed with nicotine dependence [106]. Prospective studies have also shown that smoking is a factor in chronic widespread pain [107], and an indicator of the increased recurrence of chronic pain [108]. Additionally, smokers tend to experience greater pain intensity and have more affected areas [109,110], possibly due to dysfunction in endogenous pain modulation mechanisms [111]. Physical activity and exercise stand out as effective non-pharmacological strategies to reduce tobacco use [112,113]. A meta-analysis has shown that exercise produces significant positive short-term effects on tobacco use in non-chronic pain populations, especially during the intervention period, due to changes in behavior and lifestyles, driven by affective, biological, and cognitive factors [114]. In the non-chronic pain population, exercise significantly reduces smoking satisfaction and craving by reducing the psychological reward associated with smoking, pleasurable airway sensations, and withdrawal symptoms [115,116]. It also improves self-efficacy, coping, and sleep quality, and reduces anxiety and depression [117,118,119,120]. Physiologically, exercise stimulates dopamine release, activating the limbic reward system [121] and endorphins [122], replacing the rewarding effects of tobacco and counteracting addictive behavior [123,124]. Physical activity also increases serotonin levels [125], regulates the release of GABA (an inhibitory neurotransmitter that reduces anxiety) [126], and modulates glutamate levels (an excitatory neurotransmitter whose overexcitation intensifies symptoms) [127], helping to alleviate withdrawal symptoms [128]. In summary, exercise, by enhancing neurobiological rewards, alleviating withdrawal symptoms, and reducing anxiety, is presented as a potential strategy to help people with chronic pain to quit smoking and improve their health status. Still, future research should reveal whether the promising findings from non-chronic pain populations can be translated to the chronic pain population.

**Figure 2:**
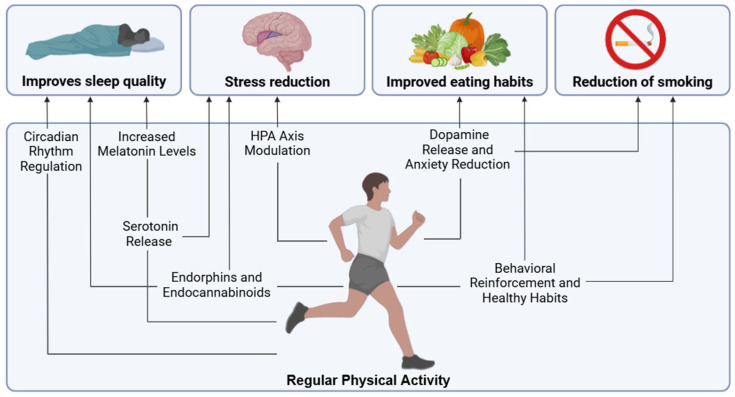
Main effects through which physical activity can influence other key healthy lifestyles for chronic pain.

The main effects through which physical activity can influence other key healthy lifestyles for chronic pain are presented in Figure 2.

A summary of representative studies included in this narrative review is presented in Table A2.

## 4. Comprehensive Intervention Proposal

**Figure 3:**
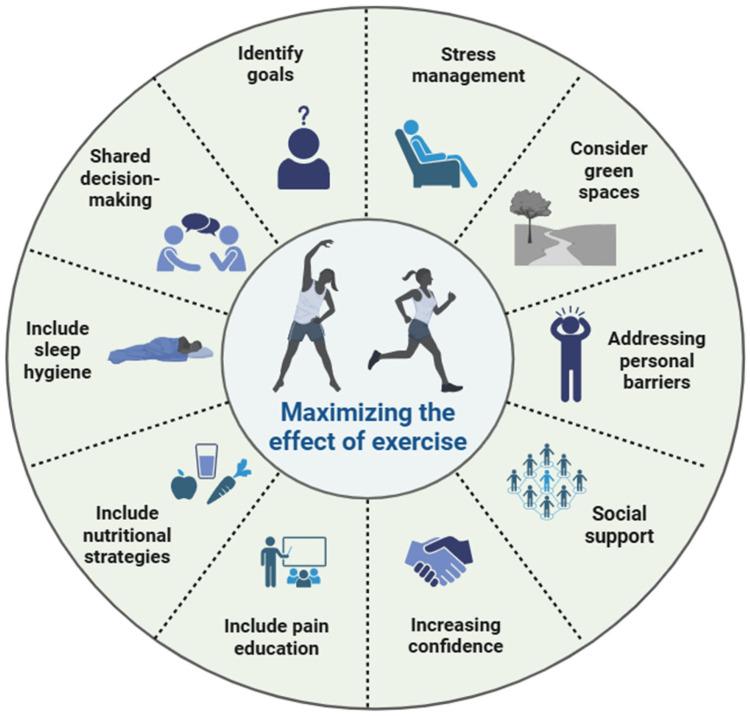
Strategies to maximize effect of exercise and physical activity in people with chronic pain.

Clinicians are advised to educate patients with chronic pain about the benefits of exercise therapy not only for pain management, but also for improving sleep, diet, and stress, encouraging sustained adherence. Furthermore, to maximize the benefits of exercise in people with chronic pain, it is essential to adopt a holistic approach which includes the following aspects:

(I) The design of individualized programs, including aerobic, resistance, and flexibility exercises, adapted to the patient’s physical and emotional capacities, taking into account their preferences and goals. Shared decision making is essential to promote adherence to exercise programs in people with chronic pain [129].

(II) Exercise should be tailored for patients with chronic pain according to their avoidance and persistence behaviors [88]. For avoided activities, it is recommended to use behavioral graded activity or exposure in vivo according to the level of perceived threat. Graded activity should focus on personal goals, progressing gradually to overcome fear of movement. In persistent activities, it is recommended to encourage self-management with breaks and acceptance strategies.

(III) The integration of exercise with pain education, nutritional strategies, sleep hygiene, and stress management to address the multiple dimensions of chronic pain, optimizing exercise therapy. Recent meta-analyses have determined that optimal doses (between 100 and 200 total minutes) of pain science education added to exercise are effective in reducing kinesiophobia, anxiety, pain intensity, and disability in people with chronic pain [130,131]. In addition, they highlight the importance of integrating pain knowledge transfer to promote adaptive changes in behavior [132]. Thus, incorporating pain education and reinforcing positive associations can increase confidence in physical activity, promoting its safe and effective integration into daily life. Improving the quality of sleep can enhance the effects of exercise, given its essential role in processes such as motor memory consolidation [133]. Cognitive–behavioral therapy for insomnia has been established as an effective intervention to treat insomnia in people with chronic pain [134]. In this regard, a 250 min dose of cognitive–behavioral therapy for insomnia has shown a great effect in reducing insomnia, reaching a maximum effect at 450 min dosed over several sessions [134]. Also, proper nutrition is key to supporting exercise, improving recovery, and preventing injuries [105].

(IV) Clinicians should be aware of the various barriers that may limit physical activity, including intrapersonal factors (e.g., beliefs and fear of movement), interpersonal factors (e.g., lack of social support), environmental concerns (e.g., environmental safety), and systemic issues (e.g., lack of knowledge and programs) [135,136,137]. To overcome these barriers, it is essential to educate patients, implement progressive programs, encourage group exercise, and develop government policies that ensure equitable and timely access to physical activity programs [138]. Strategies to maximize the effect of exercise and physical activity in people with chronic pain are summarized in Figure 3.

### Limitations

This review has several limitations that should be considered. First, the narrative nature of the review may limit the robustness of the conclusions drawn. Second, although we used a structured search strategy, the inclusion of studies was based on thematic relevance and did not involve formal risk-of-bias assessment. Third, the heterogeneity of the included studies in terms of populations, interventions, and outcome measures may affect the generalizability of the findings. Finally, the biological mechanisms discussed may reflect emerging or preclinical findings that are not yet fully validated in clinical settings.

## 5. Conclusions

Physical activity is an essential part of a comprehensive approach to chronic pain because of its ability to reduce pain, improve sleep quality, regulate metabolic processes, and manage stress. These positive effects create a virtuous circle that promotes both physical and mental health in people with chronic pain. The implementation of exercise programs should be personalized and self-directed, integrating the biopsychosocial model to address individual needs. In addition, it is important to incorporate multimodal strategies that include pain education, the progressive grading of activity, and adaptive behavior modification to promote sustained adherence. Although the available evidence is promising, more randomized controlled trials are needed to examine the effects of exercise on other healthy lifestyle behaviors, such as stress reduction, dietary modification, and smoking cessation, to consolidate its role in the comprehensive prevention and management of chronic pain.

## Data Availability

Data sharing is not applicable.

## Abbreviations

BDNF	Brain-derived neurotrophic factor
HPA	Hypothalamic–pituitary–adrenal
NMDA	N-Methyl-D-Aspartate
IL-6	Interleukin-6
IL-8	Interleukin-8
TNF-α	Tumor necrosis factor alpha
REM	Rapid eye movement
GLP-1	Glucagon-like peptide-1
CCK	Cholecystokinin
GABA	Gamma-aminobutyric acid

## Appendix A

**Table A1:** Search strategy.

Pubmed/MEDLINE	
#1	“chronic pain”[MeSH Terms] OR “chronic pain”[Title/Abstract]
#2	“physical activity”[Title/Abstract] OR “exercise”[Title/Abstract] OR “exercise”[MeSH Terms]
#3	“sleep”[MeSH Terms] OR “sleep”[Title/Abstract] OR “insomnia”[Title/Abstract] OR “stress”[Title/Abstract] OR “diet”[MeSH Terms] OR “diet”[Title/Abstract] OR “nutrition”[Title/Abstract] OR “smoking”[MeSH Terms] OR “smoking”[Title/Abstract]
#4	#1 AND #2 AND #3
#5	Filters applied: Meta-Analysis, Randomized Controlled Trial, Review, Systematic Review.
Embase	
#1	(‘chronic pain’:ab,ti)
#2	(‘physical activity’:ab,ti OR exercise:ab,ti)
#3	(sleep:ab,ti OR insomnia:ab,ti OR stress:ab,ti OR diet:ab,ti OR nutrition:ab,ti OR smoking:ab,ti)
#4	#1 AND #2 AND #3
#5	#4 AND (‘article’/it OR ‘clinical trial’/it OR ‘review’/it)
Cochrane Reviews	
#1	“chronic pain”
#2	“physical activity” OR “exercise”
#3	“sleep” OR “insomnia” OR “stress” OR “diet” OR “nutrition” OR “smoking”
#4	#1 AND #2 AND #3

**Table A2:** Summary of representative studies included.

Study	Design	Population	Topic	Type of Physical Activity	Main Findings
Chang et al. 2024 [53]	Systematic review with network meta-analysis (107 RCTs)	8121 adults with chronic musculoskeletal pain	Sleep	Aerobic exercise, resistance training, mind–body (e.g., Tai Chi)	Exercise and mind–body exercise significantly improved sleep quality.
Navarro-Ledesma et al. 2024 [55]	Systematic review (17 RCTs)	Adults with chronic musculoskeletal pain (n = 591)	Sleep	Aerobic, resistance, Pilates, mind–body	Therapeutic exercise showed positive effects on sleep quality. More research is needed to determine optimal exercise parameters.
Calvo et al. 2023 [54]	Systematic review with meta-analysis (6 RCTs, 4 in meta-analysis)	636 adults with chronic pain and sleep disturbance (mean age 54 ± 9.7)	Sleep	Neck stabilization, Pilates, walking, yoga, aerobics, supervised group exercise	Most interventions improved sleep and pain in the qualitative synthesis, but the meta-analysis showed no statistically significant effects. A correlation between pain and sleep improvements was noted.
Rotter et al. 2022 [85]	RCT	55 adults (82% women) with chronic low back pain (VAS ≥ 40 mm)	Stress	Mindful walking (8 weekly 60 min guided sessions with mindfulness training and self-practice)	No statistically or clinically significant differences were found in terms of pain, stress, or function after 8 weeks. A slight improvement tendency was noted at 12 weeks.
Aykut and Şevgin 2025 [84]	RCT	46 women (18–35) with premenstrual syndrome	Stress	Home-based Pilates (video-guided, 2x/week for 8 weeks)	Pilates significantly reduced symptoms of central sensitization, perceived stress, and pain in the intervention group compared to controls.
Verbrugghe et al. 2023 [86]	Secondary analysis of RCT	51 adults (mean age 43.6) with chronic non-specific low back pain	Stress	High-intensity interval training: aerobic + resistance/core strength training	Exercise reduced perceived stress at 6 months. Stronger effects were observed in participants with clinically elevated CSI scores. A small but significant correlation with improvements in disability and pain was observed.
Messier et al. 2013 [103]	RCT (18 months, 3 arms)	454 overweight/obese older adults (≥55 años) with knee osteoarthritis	Diet and inflammation	Aerobic + strength (3x/sem) combined with diet	The diet plus exercise group showed greater improvements in pain, function, IL-6 levels, physical quality of life, and weight loss than those with diet or exercise alone.

Abbreviations: RCT = randomized controlled trial; VAS = Visual Analog Scale; CSI = Central Sensitization Inventory.

The flowchart summarizes the records retrieved through the structured database search. Additional relevant studies identified via manual citation tracking or thematic relevance were also discussed in the narrative synthesis but are not represented in this diagram.
